# Carbapenem and Cephalosporin Resistance among *Enterobacteriaceae* in Healthcare-Associated Infections, California, USA^1^

**DOI:** 10.3201/eid2507.181938

**Published:** 2019-07

**Authors:** Kyle Rizzo, Sam Horwich-Scholefield, Erin Epson

**Affiliations:** California Department of Public Health, Richmond, California, USA.

**Keywords:** drug resistance, multidrug-resistant, bacteria, carbapenems, *Enterobacteriaceae*, enteric infections, cephalosporins, infections, hospital, epidemiology, antimicrobial resistance, California, United States

## Abstract

We analyzed antimicrobial susceptibility test results reported in healthcare-associated infections by California hospitals during 2014–2017. Approximately 3.2% of *Enterobacteriaceae* reported in healthcare-associated infections were resistant to carbapenems and 26.9% were resistant to cephalosporins. The proportion of cephalosporin-resistant *Escherichia coli* increased 7% (risk ratio 1.07, 95% CI 1.04–1.11) per year during 2014–2017.

The Centers for Disease Control and Prevention (CDC) identified carbapenem-resistant *Enterobacteriaceae* (CRE) as an urgent public health threat and extended-spectrum β-lactamase (ESBL)–producing *Enterobacteriaceae* as a serious public health threat (*1*). Antimicrobial-resistant pathogens, such as CRE, can spread across regions when infected or colonized patients transfer between healthcare facilities without infection control measures in place to prevent transmission (*2*). Therefore, tracking regional changes in antimicrobial resistance (AMR) is essential to inform public health prevention and containment strategies.

## The Study

Healthcare-associated infection (HAI) pathogen data reported to the National Healthcare Safety Network (NHSN) can be used to estimate the prevalence of AMR among hospitals within a region (*3*–*5*). Hospitals provide pathogen and antimicrobial susceptibility test results for <3 microorganisms when reporting central line–associated bloodstream infections (CLABSI), surgical site infections (SSI), and catheter-associated urinary tract infections (CAUTI) to NHSN (*6*). Data on molecular mechanisms of resistance are not collected for CLABSI, SSI, or CAUTI.

We applied CDC definitions to identify antimicrobial-resistant phenotypes among *Enterobacteriaceae*, including *Escherichia coli*, *Klebsiella* species, and *Enterobacter* species, reported in CLABSI, SSI, and CAUTI by general acute-care hospitals in California (*3*). We included multiple pathogens per HAI if reported. California hospitals report HAI data for <28 surgical procedures; we included pathogen data from any SSI reported. We excluded HAI data reported by other hospital types, such as critical access and long-term acute-care hospitals, due to limited HAI data reported by these hospitals.

According to CDC definitions, CRE were resistant to imipenem, meropenem, doripenem, or ertapenem. Extended-spectrum cephalosporin-resistant (ESCR) *Enterobacteriaceae* were resistant to ceftriaxone, ceftazidime, cefepime, or cefotaxime. We applied modified phenotype definitions from Magiorakos et al. to identify multidrug-resistant (MDR), extensively drug-resistant (XDR), and pandrug-resistant (PDR) *Enterobacteriaceae* (*7*). Susceptibility data for 2 antimicrobial drugs (ceftaroline and fosfomycin) included in these definitions were not available in our NHSN data. Resistance was defined by an isolate’s nonsusceptibility to >1 agent (e.g., imipenem) within a category of antimicrobial drugs (e.g., carbapenems) and the total number of antimicrobial categories (<15) for which the isolate was nonsusceptible. MDR *Enterobacteriaceae* were nonsusceptible to >3 antimicrobial categories; XDR *Enterobacteriaceae* were nonsusceptible to all but 1 or 2 antimicrobial categories, and PDR *Enterobacteriaceae* were nonsusceptible to all antimicrobial categories. We also assessed the phenotype difficult-to-treat (DTR) proposed by Kadri et al. (*8*). DTR included an intermediate or resistant result to all reported agents within carbapenem, cephalosporin, and fluoroquinolone categories, as well as piperacillin-tazobactam and aztreonam when results were available.

We used log binomial regression models to estimate statewide, year-to-year change in the proportion of antimicrobial-resistant *Enterobacteriaceae* during 2014–2017. To understand regional differences in CRE and ESCR *Enterobacteriaceae*, we performed a subgroup analysis in which we aggregated HAI data in 2-year increments and measured percentage resistance by county when susceptibility test results for >30 *Enterobacteriaceae* were available. CDC has explored risk adjustment for regional-level comparisons using NHSN data and determined unadjusted measures are satisfactory until additional covariates are adopted in NHSN (*9*).

We completed data analyses in SAS version 9.4 (SAS, http://www.sas.com) and spatial analyses in ArcMap version 10.4 (Environmental Systems Research Institute, Inc., https://www.esri.com). This public health surveillance analysis met criteria for nonresearch activity and did not require an exemption determination from the California Committee for the Protection of Human Subjects.

During 2014–2017, 305 (91%) of 335 California hospitals reported >1 *Enterobacteriaceae* in HAI with cephalosporin susceptibility test results; 296 (88%) hospitals reported >1 *Enterobacteriaceae* with carbapenem susceptibility test results. The median number of *Enterobacteriaceae* reported with cephalosporin susceptibility test results by hospitals per year was 8 (interquartile range 16–3), and 6 (interquartile range 14–3) for *Enterobacteriaceae* with carbapenem susceptibility test results.

Approximately 3.2% of *Enterobacteriaceae* reported in HAI during 2014–2017 were resistant to carbapenems and 26.9% of *Enterobacteriaceae* reported in HAI were cephalosporin resistant. We observed increases in the proportions of *Enterobacteriaceae* that were ESCR and MDR during 2014–2017; these changes were driven by *E. coli* (Table 1). We observed a 7% (risk ratio [RR] 1.07; 95% CI 1.04–1.11) annual increase in the proportion of *E. coli* resistant to cephalosporins and a 4% (RR 1.04; 95% CI 1.02–1.06) annual increase in the proportion of *E. coli* with an MDR phenotype during 2014–2017 (Table 1). The proportion of *E. coli* exhibiting carbapenem resistance also increased 24% (RR 1.24; 95% CI 1.00–1.56) per year during 2014–2017.

**Table 1:** Carbapenem and cephalosporin resistance among *Enterobacteriaceae* reported in healthcare-associated infections by California hospitals, 2014–2017*

Antimicrobial agent	2014			2015			2016			2017			Change	
	No. (%) isolates†	% R		No. (%) isolates†	% R		No. (%) isolates†	% R		No. (%) isolates†	% R		Risk ratio (95% CI)	p value
*Enterobacteriaceae*														
Carbapenems	2,747 (60.2)	3.1		3,310 (64.2)	3.1		3,409 (64.2)	3.5		3,247 (65.1)	3.0		1.00 (0.92–1.09)	0.98
Cephalosporins	3,303 (74.3)	24.0		3,837 (76.2)	27.7		4,020 (77.4)	27.5		3,885 (79.6)	28.0		1.04 (1.02–1.07)	0.001
DTR	2,298 (50.0)	2.2		2,786 (53.5)	2.0		2,916 (54.5)	2.1		2,856 (56.6)	1.6		0.92 (0.81–1.04)	0.16
MDR	4,500 (98.0)	38.8		5,129 (98.5)	43.3		5,228 (97.6)	43.8		4,942 (97.9)	44.0		1.04 (1.02–1.05)	<0.001

*Escherichia coli*														
Carbapenems	1,623 (59.9)	0.7		1,969 (64.9)	0.7		1,969 (64.6)	1.1		1,893 (66.7)	1.2		1.24 (1.00–1.56)	0.05
Cephalosporins	1,890 (71.1)	22.9		2,158 (72.4)	28.0		2,229 (74.2)	27.1		2,147 (76.8)	29.7		1.07 (1.04–1.11)	<0.001
DTR	1,323 (48.6)	0.5		1,577 (51.7)	0.3		1,615 (52.7)	0.5		1,613 (56.3)	0.4		0.99 (0.69–1.42)	0.95
MDR	2,669 (98.0)	42.8		3,004 (98.4)	47.0		2,998 (97.8)	47.1		2,812 (98.2)	49.2		1.04 (1.02–1.06)	<0.001

*Enterobacter *spp.														
Carbapenems	489 (62.1)	3.7		550 (62.9)	6.9		602 (63.7)	5.2		559 (63.0)	5.4		1.06 (0.90–1.24)	0.51
Cephalosporins	701 (94.1)	29.8		786 (94.5)	30.9		855 (94.9)	33.6		811 (94.5)	30.8		1.02 (0.97–1.07)	0.47
DTR	488 (60.6)	0.2		554 (61.8)	0.5		608 (63.1)	0.5		564 (61.2)	0		0.79 (0.38–1.57)	0.50
MDR	789 (98.0)	43.5		883 (98.5)	53.0		940 (97.5)	55.6		897 (97.3)	50.3		1.04 (1.01–1.07)	0.01

*Klebsiella *spp.														
Carbapenems	635 (59.7)	8.8		791 (63.2)	6.6		838 (63.8)	7.9		795 (63.3)	5.7		0.90 (0.80–1.01)	0.07
Cephalosporins	712 (68.3)	21.4		893 (73.1)	24.2		936 (72.6)	22.8		927 (75.5)	21.5		0.99 (0.94–1.05)	0.76
DTR	487 (45.7)	8.8		655 (52.1)	7.2		693 (52.4)	7.2		679 (53.8)	5.7		0.88 (0.77–1.00)	0.06
MDR	1,042 (97.8)	25.1		1,242 (98.7)	27.7		1,290 (97.5)	27.2		1,233 (97.7)	27.8		1.03 (0.99–1.07)	0.21

*Carbapenem-resistant *Enterobacteriaceae *were resistant to imipenem, meropenem, doripenem, or ertapenem. *Enterobacteriaceae *resistant to ceftriaxone, ceftazidime, cefepime, or cefotaxime were cephalosporin-resistant. MDR *Enterobacteriaceae *were nonsusceptible to >3 antimicrobial categories; XDR *Enterobacteriaceae *were nonsusceptible to all but 1 or 2 antimicrobial categories and pandrug-resistant *Enterobacteriaceae *were nonsusceptible to all antimicrobial categories (n = 15). DTR *Enterobacteriaceae *were intermediate or resistant to all reported agents within carbapenem, cephalosporin, and fluoroquinolone categories, as well as piperacillin-tazobactam and aztreonam when results were available. DTR, difficult-to-treat; MDR, multidrug-resistant; % R, percentage resistant. †The number and percentage of *Enterobacteriaceae *with antimicrobial susceptibility test results as a proportion of the overall number reported (i.e., with or without antimicrobial susceptibility test results).

We observed decreasing trends in carbapenem resistance (RR 0.90; 95% CI 0.80–1.01) and in the DTR phenotype (RR 0.88; 95% CI 0.77–1.00) among *Klebsiella* species reported in HAI. Among *Enterobacteriaceae* assessed for the DTR phenotype, *Klebsiella* species accounted for 86% (n = 193) of DTR isolates and comprised 23% of the overall total of *Enterobacteriaceae* analyzed among HAI. In addition, 1 XDR *Klebsiella pneumoniae* was reported in HAI during 2014–2017 and no PDR *Enterobacteriaceae* were reported.

**Figure 1:**
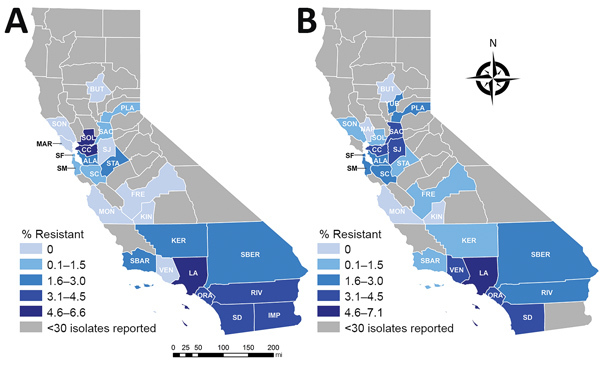
Geographic distribution of carbapenem resistance among *Enterobacteriaceae* reported in healthcare-associated infections by hospitals, aggregated by county, California, 2014–2015 (A) and 2016–2017 (B). ALA, Alameda; BUT, Butte; CC, Contra Costa; FRE, Fresno; IMP, Imperial; KER, Kern; KIN, Kings; LA, Los Angeles; MAR, Marin; MON, Monterey; NAP, Napa; ORA, Orange; PLA, Placer; RIV, Riverside; SAC, Sacramento; SBER, San Bernardino; SD, San Diego; SF, San Francisco; SJ, San Joaquin; SM, San Mateo; SBAR, Santa Barbara; SC, Santa Clara; SOL, Solano; SON, Sonoma; STA, Stanislaus; VEN, Ventura; YUB, Yuba.

**Figure 2:**
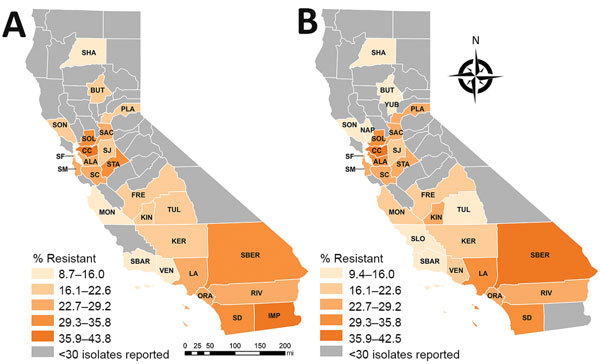
Geographic distribution of cephalosporin resistance among *Enterobacteriaceae* reported in healthcare-associated infections by hospitals, aggregated by county, California, 2014–2015 (A) and 2016–2017 (B). ALA, Alameda; BUT, Butte; CC, Contra Costa; FRE, Fresno; IMP, Imperial; KER, Kern; KIN, Kings; LA, Los Angeles; MON, Monterey; NAP, Napa; ORA, Orange; PLA, Placer; RIV, Riverside; SAC, Sacramento; SBER, San Bernardino; SD, San Diego; SF, San Francisco; SJ, San Joaquin; SLO, San Luis Obispo; SM, San Mateo; SBAR, Santa Barbara; SC, Santa Clara; SHA, Shasta; SOL, Solano; SON, Sonoma; STA, Stanislaus; TUL, Tulare; VEN, Ventura; YUB, Yuba.

Percentages of CRE and ESCR phenotypes varied by county and reporting years (Table 2; Figures 1, 2). Carbapenem and cephalosporin resistance was higher in California regions more densely populated with hospitals and residents, such as the greater Los Angeles region and San Francisco Bay area. Counties with hospitals reporting <30 *Enterobacteriaceae* may still have antimicrobial-resistant HAI or receive patients from healthcare facilities where antimicrobial resistance is endemic.

**Table 2:** Carbapenem and cephalosporin resistance among *Enterobacteriaceae* reported in healthcare-associated infections by California hospitals, aggregated by county, 2014–2017*

County	Carbapenems						Cephalosporins				
	2014–2015			2016–2017			2014–2015			2016–2017	
	No. (%) isolates†	% R		No. (%) isolates†	% R		No. (%) isolates†	% R		No. (%) isolates†	% R
Alameda	280 (72.4)	1.8		342 (84)	2.0		215 (56.3)	27.4		258 (63.2)	29.8
Butte	40 (97.6)	0		46 (93.9)	0		40 (100)	22.5		47 (97.9)	14.9
Contra Costa	141 (50.5)	5.7		197 (57.8)	3.0		120 (43.8)	40.0		159 (46.9)	40.3
Fresno	324 (91.3)	0		379 (95.5)	0.3		293 (85.4)	18.4		351 (89.5)	21.1
Imperial	33 (100)	3.0		NS	NS		32 (97.0)	43.8		NS	NS
Kern	165 (97.6)	1.8		137 (89)	0.7		100 (61.0)	17.0		110 (71.9)	19.1
Kings	35 (100)	0		33 (100)	0		36 (100)	16.7		33 (100)	27.3
Los Angeles	1,294 (46.9)	6.6		1,477 (49.6)	7.1		2,044 (74.7)	28.7		2,263 (75.8)	32.4
Marin	38 (97.4)	0		NS	NS		NS	NS		NS	NS
Monterey	85 (95.5)	0		111 (78.7)	0		69 (76.7)	8.7		112 (80.6)	16.1
Napa	NS	NS		31 (93.9)	0		NS	NS		32 (97.0)	9.4
Orange	365 (56.6)	3.6		363 (54.7)	4.1		530 (85.8)	26.4		548 (84.8)	24.5
Placer	70 (70.0)	1.4		108 (89.3)	1.9		60 (60.0)	21.7		79 (64.8)	24.1
Riverside	233 (56.0)	3.4		249 (55.8)	2.4		315 (77.2)	23.8		371 (84.1)	22.6
Sacramento	381 (86.6)	1.0		392 (81.8)	3.3		336 (78.1)	22.9		388 (82.9)	27.3
San Bernardino	301 (67.9)	3.0		337 (72.9)	3.0		217 (61.8)	33.2		212 (67.9)	42.5
San Diego	657 (53.8)	4.0		573 (50.8)	3.1		946 (80.0)	32.7		901 (81.5)	31.7
San Francisco	405 (96.4)	1.2		432 (96.0)	2.1		374 (87.4)	21.7		381 (84.1)	22.6
San Joaquin	61 (71.8)	0		53 (54.1)	3.8		80 (94.1)	16.3		95 (97.9)	18.9
San Luis Obispo	NS	NS		NS	NS		NS	NS		30 (100)	13.3
San Mateo	137 (98.6)	0.7		107 (98.2)	2.8		92 (67.2)	28.3		82 (73.9)	25.6
Santa Barbara	113 (99.1)	1.8		106 (98.1)	0.9		108 (95.6)	11.1		107 (99.1)	11.2
Santa Clara	370 (55.2)	1.4		557 (69.2)	1.8		413 (62.5)	25.9		659 (81.1)	26.9
Shasta	NS	NS		NS	NS		67 (97.1)	11.9		59 (96.7)	10.2
Solano	91 (95.8)	6.6		121 (99.2)	0.8		55 (58.5)	32.7		80 (67.2)	35.0
Sonoma	71 (68.3)	0		99 (92.5)	1.0		81 (78.6)	17.3		77 (72.6)	14.3
Stanislaus	92 (62.2)	2.2		98 (66.2)	1.0		106 (74.6)	29.2		98 (68.1)	28.6
Tulare	NS	NS		NS	NS		36 (69.2)	22.2		41 (74.5)	14.6
Ventura	42 (29.0)	0		61 (48.4)	3.3		128 (89.5)	11.7		119 (95.2)	20.2
Yuba	NS	NS		36 (97.3)	2.8		NS	27.4		37 (97.4)	13.5

*Carbapenem-resistant *Enterobacteriaceae* were resistant to imipenem, meropenem, doripenem, or ertapenem. *Enterobacteriaceae* resistant to ceftriaxone, ceftazidime, cefepime, or cefotaxime were cephalosporin resistant. The percentage of resistant *Enterobacteriaceae* is not shown when <30 *Enterobacteriaceae *are reported within a county. NS, not shown; % R, percentage resistant. †The number and percentage of *Enterobacteriaceae *with reported antimicrobial susceptibility test results as a proportion of the overall number reported (i.e., with or without antimicrobial susceptibility test results).

Several factors limit the interpretation of our results. Only 4 years of data were available for measuring AMR trends. Selective reporting of susceptibility test results may have restricted sample sizes and increased the potential for sampling bias to affect our results. Furthermore, there may be differences in how California hospitals and laboratories interpret MIC breakpoints or changes in how breakpoints are applied over time. Data on molecular mechanisms of resistance are not collected in CLABSI, SSI, or CAUTI, which limits our understanding of how transmissible elements, including ESBL and carbapenemases, may contribute to the trends we observed.

## Conclusions

Increases in carbapenem, cephalosporin, and MDR *E. coli* reported in HAI by California hospitals are concerning, given that *E. coli* are common causes of both hospital and community-associated infections. ESBL-producing *E. coli* have been reported in community-associated urinary tract infections among patients in California, with estimates of resistance among *E. coli* from 5% up to 17% in complicated pyelonephritis (*10*,*11*). MDR and DTR *Enterobacteriaceae* further limit treatment options and present management challenges, particularly in outpatient settings when there are no oral antimicrobial treatment options.

AMR prevention and containment strategies may depend on the local prevalence. For example, prompt detection and rapid, aggressive containment responses to individual AMR cases can be effective in low-prevalence regions. Admission screening and empiric use of transmission-based precautions for patients at high risk for AMR might be more feasible in higher-prevalence regions.

Healthcare facilities can prevent HAI and the spread of AMR by implementing best practices in infection control and antimicrobial stewardship. State and local health departments can coordinate prevention efforts across the healthcare continuum, investigate and control outbreaks in healthcare facilities, and set expectations for healthcare facilities to communicate patients’ AMR infection and colonization status during all patient transfers. Decreasing trends in carbapenem resistance and in the DTR phenotype among *Klebsiella* species, often the focus of AMR containment efforts, indicate the potential effectiveness of such prevention strategies (*5*). Nonetheless, increases and regional variation in carbapenem-resistant and ESCR *E. coli* highlight the urgent need for ongoing, local infection prevention and antimicrobial stewardship efforts.
